# Whipped oil stabilised by surfactant crystals†
†Electronic supplementary information (ESI) available. See DOI: 10.1039/c6sc00046k


**DOI:** 10.1039/c6sc00046k

**Published:** 2016-03-03

**Authors:** Bernard P. Binks, Emma J. Garvey, Josélio Vieira

**Affiliations:** a Department of Chemistry , University of Hull , Hull , HU6 7RX , UK . Email: b.p.binks@hull.ac.uk; b Nestlé Product Technology Centre , PO Box 204, Haxby Road , York , YO91 1XY , UK

## Abstract

We describe a protocol for preparing very stable air-in-oil foams starting with a one-phase oil solution of a fatty acid (myristic acid) in high oleic sunflower oil at high temperature. Upon cooling below the solubility limit, a two-phase mixture consisting of fatty acid crystals (length around 50 μm) dispersed in an oil solution at its solubility is formed which, after whipping, coat air bubbles in the foam. Foams which do not drain, coalesce or coarsen may be produced either by increasing the fatty acid concentration at fixed temperature or aerating the mixtures at different temperatures at constant concentration. We prove that molecular fatty acid is not surface-active as no foam is possible in the one-phase region. Once the two-phase region is reached, fatty acid crystals are shown to be surface-active enabling foam formation, and excess crystals serve to gel the continuous oil phase enhancing foam stability. A combination of rheology, X-ray diffraction and pulsed nuclear magnetic resonance is used to characterise the crystals and oil gels formed before aeration. The crystal-stabilised foams are temperature-sensitive, being rendered completely unstable on heating around the melting temperature of the crystals. The findings are extended to a range of vegetable oil foams stabilised by a combination of adsorbed crystals and gelling of the oil phase, which destabilise at different temperatures depending on the composition and type of fatty acid chains in the triglyceride molecules.

## Abstract 2

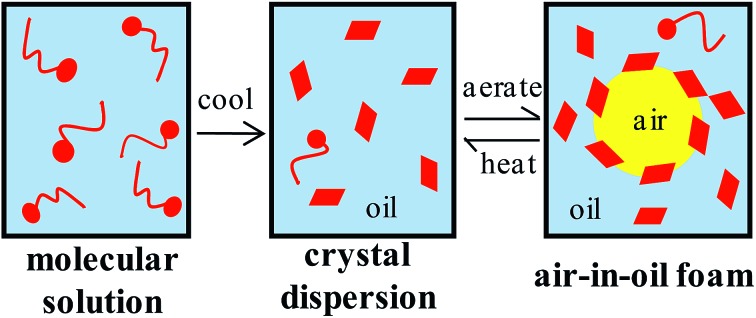
We describe a protocol for preparing very stable air-in-oil foams containing surfactant crystals starting from a one-phase solution of a fatty acid in a vegetable oil. On cooling, plate-like crystals form which adsorb at air bubble surfaces after aeration of the mixture. Such foams can be destabilised by gradual warming leading to crystal melting and bubble coalescence.

## Introduction

There is scant literature on the foaming of oils, *i.e.* gas-in-oil, in sharp contrast to that of aqueous-based foams. This is despite their occurrence in a number of industries, including the food and oilfield chemical ones. For example, bubbles are included in chocolate (fat-based) not to add any nutritional value but to change the texture and mouth-feel characteristics of importance to the consumer.^1^ Such foams are lighter than the oil alone and of current interest in foods due to the reduced oil/fat content. The unwanted foaming of petroleum or crude oil is a major problem during gas–oil separation and appropriate antifoams need to be designed which involve specific considerations not encountered in aqueous systems.^2^–^4^ Since the surface tension of most hydrocarbon-containing oils is low (<35 mN m^–1^) compared with that for water (72 mN m^–1^ at 25 °C), the driving force for adsorption of many hydrocarbon-based surfactants to their surface is significantly reduced.^5^

Nonetheless, foam stabilisation of oils has been achieved using a number of strategies. Early work by Ross and co-workers with lubricating oils demonstrated the importance of nearness of the system to the solubility phase boundary of the added foaming agent in the oil.^6^,^7^ Foamability and foam stability increased dramatically close to the condition where the foaming agent became insoluble and probably more surface-active. This was supported by the data of Friberg *et al.*^8^ for xylene foams stabilised by triethanolammonium oleate surfactant; no foam was produced when the surfactant formed an isotropic liquid phase but relatively stable foams occurred in the two-phase region containing a lamellar liquid crystalline phase. It was suggested that this aggregated phase adhered to air bubble surfaces. More recent work with poly(decene) oil and a range of low molar mass and polymeric surfactants has provided further evidence of foaming being enhanced around the solubility limit of a particular ingredient.^9^ Additionally, in that work, the increased viscosity of the oil phase at higher surfactant concentrations stabilised the foams to gravity-induced drainage of oil. A comprehensive series of papers by Shrestha and co-workers demonstrated that very stable foams of either liquid paraffin, squalane, squalene, a branched C_8_ triglyceride and olive oil with various long chain fatty acid esters as stabilisers could be produced depending on the location of the mixture within the phase diagram.^10^–^13^ Liquid petroleum gas was first dissolved in oil and foam was generated upon releasing the pressure in the vessel. The authors consistent conclusion was that the most stable foams to coalescence occurred when solid particles of surfactant (L_β_ phase) were present compared with aggregates of the lamellar liquid crystalline phase (L_α_ phase), and that these coated air bubbles resisted coalescence. Since the surface energy of fluorocarbons is lower than that of hydrocarbons, another strategy discussed by Bergeron *et al.*^14^ was to use fluorocarbon surfactants capable of lowering the dodecane-air tension by up to 5 mN m^–1^. Foams and foam films (air-oil-air) were stabilised *via* surfactant adsorption, with a repulsive disjoining pressure created by the overlap of surfactant layers on neighbouring bubble surfaces leading to stability.

An alternative to using molecular stabilisers for oil foams is to design suitably surface-active solid particles of low surface energy. Since particles of a suitable size range are effectively irreversibly adsorbed, ultra-stable foams should result. The idea was shown by Murakami and Bismarck^15^ for oligotetrafluoroethylene particles and by ourselves for a range of commercial polytetrafluoroethylene particles.^16^ In subsequent work, we identified the conditions required for successful foaming of a range of oils in terms of the extent of fluorination of the particles and the oil surface tension. This was exemplified for fluorinated fumed silica,^17^ fluorinated sericite clay^18^ and a range of other fluorinated particles.^19^

We report here on the whipping of vegetable oils, mainly high oleic sunflower oil (HOSO), in the presence of crystals of pure myristic acid surfactant (C_13_H_27_COOH, MA) formed *in situ* following cooling of an oil solution. The foams produced are primarily stabilised by the adsorption of the crystals around the air bubbles. We exploit the ability of non-adsorbed crystals present in the oil phase to cause its gelling, thus reducing the possibility of oil drainage and subsequent foam breakdown. An otherwise stable foam prepared at room temperature is shown to be temperature-sensitive and can be destabilised upon heating causing surfactant crystals to melt and bubbles to coalesce. Our initial findings were reported in ref. 20 and a full account including the behaviour of other model systems appeared later.^21^ Stabilisation of rapeseed oil foams by crystals of a commercial surfactant containing a mixture of mono- and diglycerides was reported recently.^22^ The study however focused on the rheology of the oil phase before foaming and that of the subsequent foam with little mention of the shape, size or structure of the crystals. As alluded to earlier, the viscosity of oils can be increased above a certain concentration of additive and air bubbles may be suspended in the rigid network so produced preventing their upward creaming thus contributing to enhanced foam stabilisation. In the case of fat crystals, fat crystal networks develop,^23^ otherwise so-called organogels^24^,^25^ are formed in which small crystals interact through van der Waals forces and intermolecular hydrogen bonds to immobilise the liquid oil. Traditionally, the common way to provide texture to the oil phase of food products has been to include a triglyceride capable of crystallising. However, these hardstocks contain saturated fatty acids which are considered unhealthy. Pernetti *et al.*^26^ review the possible alternatives investigated so far for the structuring of edible oil. A variety of surfactants has been studied including long chain alcohols and acids,^27^–^29^ 12-hydroxystearic acid,^30^ 12-hydroxyoleic acid,^31^ monoglycerides,^32^–^34^ diglycerides^35^ and triglycerides^36^ in oils like soybean, sunflower, canola, olive, palm and cod liver. The gels have been characterised using rheology, diffraction, calorimetry and microscopy. In most cases, less than 4 wt% of structurant is required to form a stiff gel. The crystal shape varies from plates to needles to ribbons.

## Results and discussion

We first present the characterisation of solutions and gels of myristic acid in HOSO with emphasis on the prevailing temperature. This is followed by a discussion of the whipping of these mixtures and the influence of temperature on destabilising an otherwise stable foam. The effects of both MA concentration at fixed temperature and aeration temperature at fixed MA concentration are evaluated. Finally, the generic nature of our findings is probed by investigating the aeration of eight other vegetable oils also stabilised by MA crystals.

### Crystal and gel formation of MA in HOSO

#### Solubility–temperature relationship

**Fig. 1:**
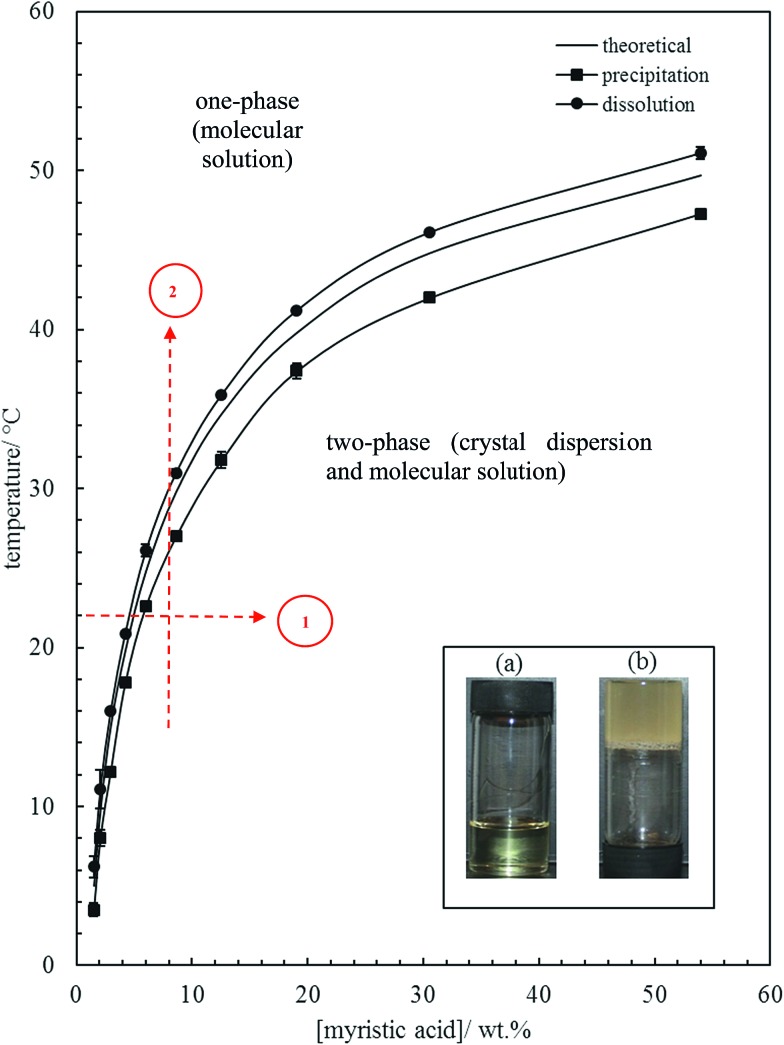
Variation of MA solubility in HOSO with temperature, with points obtained from cooling at 0.1 °C min^–1^ (squares) and heating at 0.1 °C min^–1^ (circles). The full line without points is that calculated using eqn (1). Inset: photographs of (a) oil solution, upright at 40 °C, and (b) oil gel, inverted at 22 °C, for 10 wt% MA. Routes 1 and 2 are those followed during aeration (later).

The effect of temperature on the solubility of MA in HOSO was determined primarily using visual observations. Neat MA melts at 53.5 °C and HOSO remains liquid down to at least –20 °C. Solid MA at different concentrations was dissolved in HOSO at 80 °C and left at this temperature for 10 min resulting in a clear homogeneous liquid. This one-phase solution contains MA molecules. The solution was cooled at a rate of 0.1 °C min^–1^ until the first crystal appeared at the precipitation temperature giving rise to a turbid dispersion. This two-phase mixture contains a dispersion of MA crystals in equilibrium with a molecular solution at its solubility. Upon cooling a further 5 °C and holding for 5 min, the dispersion was heated at 0.1 °C min^–1^ until the onset of clearing at the dissolution temperature. The variation of these two temperatures with MA concentration is shown in Fig. 1. As seen we observe a hysteresis of a few °C in measurements of the solubility boundary temperature by cooling and heating. Since the solubility boundary is an equilibrium quantity, the hysteresis must result from the kinetics of either crystal formation or dissolution being slow relative to the rate of temperature change used here. Crystal formation by cooling occurs by nucleation and growth requiring an energy barrier to be overcome and can be slow. Crystal dissolution by heating is expected to involve no energy barrier and is likely to be fast. Hence, the dissolution temperature should be closer to the equilibrium temperature of the solubility boundary. If we assume that the fatty acid–oil mixture forms an ideal solution and that the MA crystals contain no co-crystallised solvent, the variation of solubility with temperature is given by^37^
1

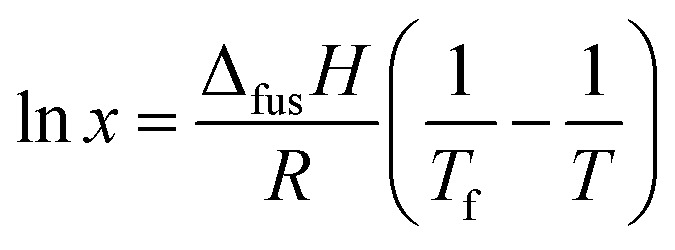

where *x* is the mole fraction of solute in the saturated solution at absolute temperature *T*, *R* is the gas constant, Δ_fus_*H* is the enthalpy of fusion for MA (45 kJ mol^–1^)^38^ and *T*_f_ is its temperature of fusion (326.6 K).^38^ Taking the relative molar mass of HOSO to be 912 g mol^–1^, eqn (1) was used to calculate the variation of MA solubility with temperature shown as the line without points in Fig. 1. It lies closer to the experimental points of the dissolution temperature as opposed to those of the precipitation temperature as expected although deviations are observed at higher concentrations. Since HOSO is not a pure oil, these may be due to slight non-ideality of the MA–HOSO mixture. Differential scanning calorimetry (DSC) was also used to investigate the thermal transitions mentioned above. The protocol consisted of cooling a mixture from 80 °C to –20 °C at different rates, holding at this temperature for 5 min before heating to 80 °C at different rates. All DSC curves displayed a single peak on cooling and a single peak on heating with hysteresis again being apparent. The temperatures corresponding to the maximum/minimum heat flow in both peaks are plotted as a function of MA concentration in Fig. S1† at two different cooling/heating rates, where (a) is following cooling and (b) is subsequent heating. An increase in cooling rate causes a decrease in the precipitation temperature and in increase in the dissolution temperature. For both temperatures however, the slowest rate of 0.1 °C results in transition temperatures closer to that calculated for ideal mixing. Higher cooling rates are known to induce the formation of smaller crystals affecting the microstructure of the oil gel containing them.^36^ Unless stated otherwise, further investigation of the oil gels involved cooling a mixture from 80 °C to 8 ± 2 °C by placing it in a freezer for 1 h (cooling rate ≈ 1 °C min^–1^) after which it was allowed to warm naturally to room temperature.

**Fig. 2:**
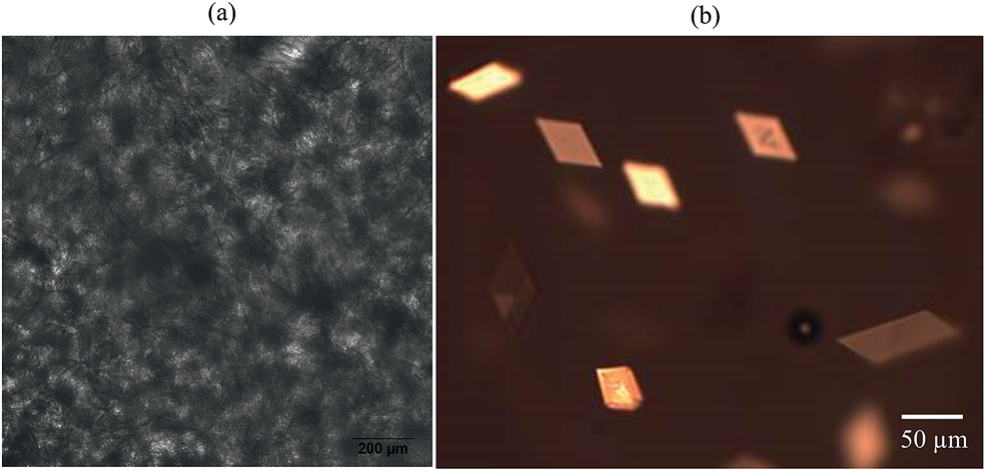
Optical microscopy images of 8 wt% MA in HOSO gel prepared at 22 °C for (a) undiluted and (b) diluted with HOSO samples.

The inset in Fig. 1 contains a photograph of a 10 wt% MA in HOSO mixture at 40 °C (a) and at 22 °C (b). In (a), the mixture is in the one-phase region and in (b) it is in the two-phase region, where at this relatively high concentration, a gel is formed which does not flow upon inverting the vessel. Optical micrographs with crossed polarisers of an oil gel prepared at 8 wt% MA are given in Fig. 2. The undiluted gel in (a) reveals a dense concentration of plate-like crystals whose side lengths are around 50 μm. By dilution with HOSO, individual plate-like crystals with prismatic shape^39^ and an acute angle of *ca.* 54° are revealed in (b) of average length around 50 μm and thickness of several microns. The two dashed arrows in Fig. 1 represent the paths followed upon aerating these mixtures as a function of MA concentration at 22 °C (path 1) and as a function of aeration temperature at 8 wt% MA (path 2) to be discussed below.

#### Crystal structure and rheology of gel

X-ray diffraction was used to determine the structure of MA crystals in HOSO at 20 ± 2 °C. The experiments were conducted on both an oil gel containing 10 wt% MA and on a crystal aggregate extracted from the same oil gel. Fig. S2† shows the X-ray pattern of the oil gel with a diffractometer employing CuKα radiation (*λ* = 1.5406 Å). A very broad feature due to diffuse scattering from the gel has been subtracted from the pattern. Three peaks due to the crystalline component are clear in the region 2 ≤ 2*θ*/° ≤ 10 and these can be indexed using the crystal structure for MA reported by Bond^40^ as shown in Table 1. The extracted MA crystal from the gel can be seen in Fig. S3(a).† The 2-D powder diffraction pattern from it employing a diffractometer using MoKα radiation (*λ* = 0.7107 Å) is given in Fig. S3(b).† In this pattern, Bragg reflections occur as rings on the image. Three rings are present in the pattern and these can also be indexed using the crystal structure for MA reported by Bond^40^ as shown in Table 1. It is clear that the structure of extracted MA crystals is the same as that of MA crystals within the gel. This carboxylic acid crystallises as the C form (monoclinic) in which all the molecules adopt an all-trans conformation. The molecules form hydrogen-bonded dimers (through –COOH) arranged into bilayers, with a rectangular packing arrangement in the plane perpendicular to the dimer long axes.^39^,^40^

**Table 1:** Characteristics of peaks expected (from ref. 40) and observed in XRD experiments. First 3 entries refer to 10 wt% MA in HOSO gel (CuKα radiation), second 3 entries refer to crystal extracted from same gel (MoKα radiation)

2*θ*/° (expected)	*d*-Spacing (expected)/Å	*I*/*I*_max_/%	2*θ*/° (observed)	Miller index
2.806	31.46	100.00	2.84	(100)
5.613	15.73	2.93	5.66	(200)
8.424	10.49	7.38	8.43	(300)

2*θ*/° (expected)	*d*-Spacing (expected)/Å	*I*/*I*_max_/%	*d*-Spacing (observed)/Å	Miller index
3.876	10.49	10.21	10.61	(300)
9.918	4.10	100.00	4.12	(3[combining macron]11)
11.176	3.64	31.03	3.65	(6[combining macron]02)

**Fig. 3:**
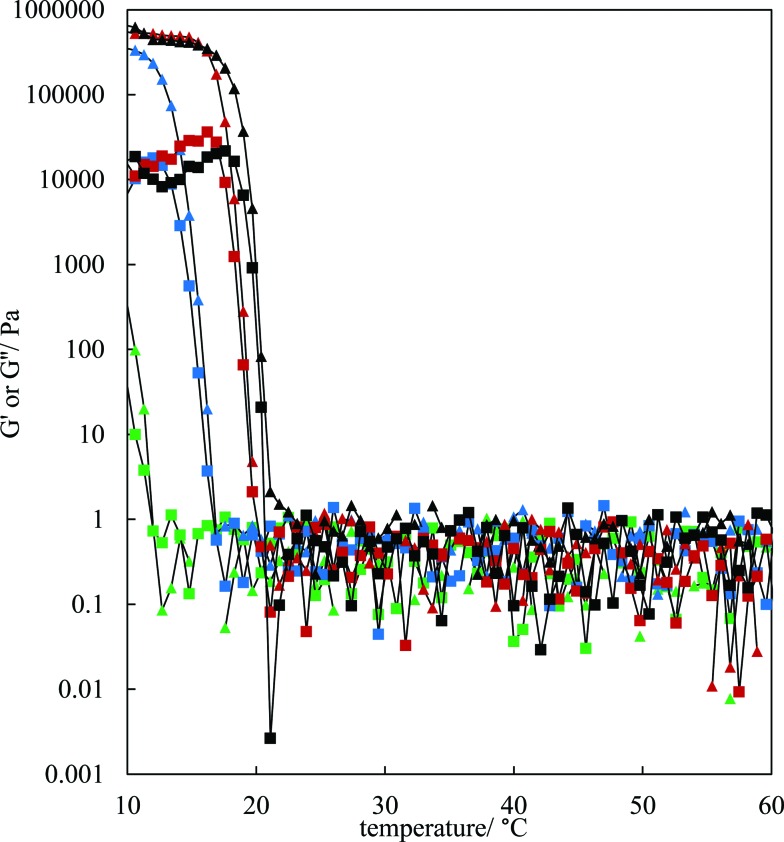
Variation of *G*′ (triangles) and *G*′′ (squares) with temperature for HOSO gels containing different concentrations of MA ((green-4 wt%, blue-5 wt%, red-6 wt%, black-7 wt%)) upon cooling from 80 °C at 1 °C min^–1^. Measurements were made at a strain of 0.01% and a frequency of 1 Hz: data only shown from 60 °C.

The rheological properties of the MA–HOSO solutions and gels were investigated using a parallel plate geometry. Initial tests were carried out to identify the viscoelastic region of 8 and 12 wt% MA in HOSO gels at 10 °C by increasing the strain from 0.001 to 1% at a fixed frequency of 1 Hz (not shown). The linear viscoelastic region was around 0.01% strain as both the elastic modulus *G*′ and the viscous modulus *G*′′ were independent of strain. At a strain of 0.01%, the effect of oscillation frequency on the magnitude of *G*′ can be seen in Fig. S4(a)† for three concentrations of MA. The value of *G*′ remains constant within this frequency range, characteristic of gelled networks,^41^ but depends on the concentration of MA. For the study of the rheology of solutions/gels at different temperatures, we selected a frequency of 1 Hz. Here, solutions at 80 °C were cooled to 10 °C at a rate of 1 °C min^–1^ for MA concentrations between 4 and 12 wt%. The variation in both *G*′ and *G*′′ with temperature for the lower concentrations is shown in Fig. 3; the corresponding plots for higher concentrations are given in Fig. S4(b).† For any concentration, values of *G*′ and *G*′′ are low around 0.05–1 Pa at relatively high temperatures (above 30 °C) and *G*′′ > *G*′ corresponding to viscous, liquid-like solutions (no crystals). At a certain temperature dependent on MA concentration, the two moduli exhibit an abrupt increase to values approaching 10^6^ Pa for 7 wt% MA at 10 °C. This corresponds to the onset and further development of crystallisation yielding oil gels where *G*′ > *G*′′, consistent with elastic, solid-like dispersions. More elastic gels are formed as the concentration of MA increases. We have extracted both the temperature midway between that giving the lowest and that giving the highest values of *G*′, and the temperature at which *G*′ reaches a maximum, from the curves in Fig. 3 and these are plotted against MA concentration in Fig. S5.† Both temperatures increase progressively with concentration, and mirror the behaviour seen earlier from visual determinations of the appearance of crystals on cooling, albeit for a different cooling rate. In any case, it is expected that the transitions with respect to temperature will be different in the two sets of experiments as a certain weight fraction of crystals is required before a gel network is formed.

#### Crystal content of gel

Pulsed nuclear magnetic resonance (NMR) spectroscopy has been used to determine the solid content (due entirely to MA crystals) in oil gels at different temperatures and as a function of MA concentration. The method is based on the fact that the NMR signal produced by a solid or a liquid, after a 90° radio frequency pulse, is proportional to the number of hydrogen atoms present in the sample.^42^,^43^ In a solid–liquid mixture, the signal is the sum of a component from the solid and one from the liquid. Due to the rapid decay of the signal from the solid compared with that from the liquid, the amount of solid and liquid in the sample can be deduced by measuring the intensity of the signal at different times after the initial pulse. The solid content (wt%) is determined using the following equation

**Fig. 4:**
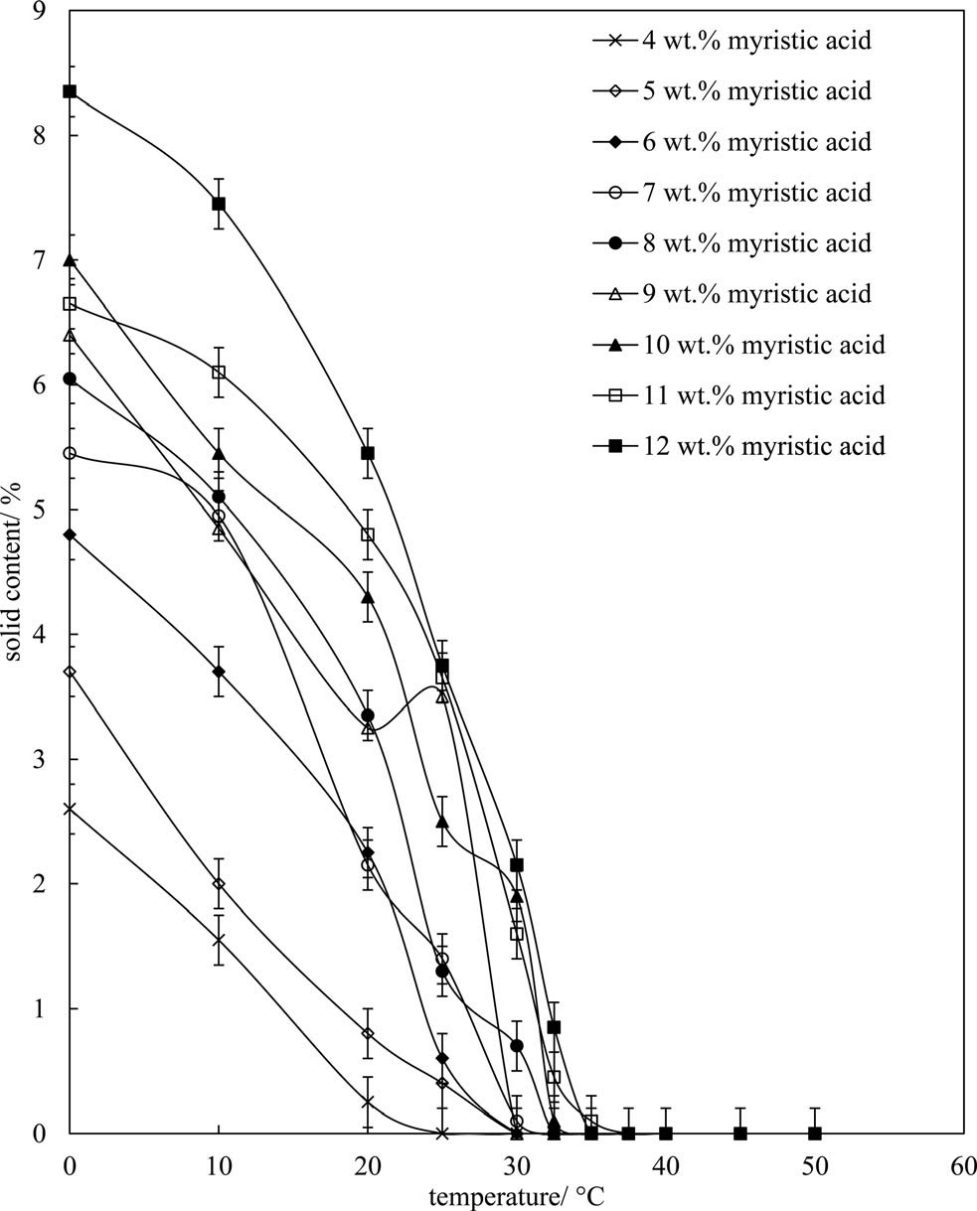
Solid content (wt%) of mixtures of MA in HOSO as a function of temperature upon cooling from 80 °C at 1 °C min^–1^ for different concentrations of MA (given). Each sample was held at the respective temperature for 30 min.

2 solid content = 100(*S*_1_ – *S*_2_)*F*/[(*S*_1_ – *S*_2_)*F* + *S*_2_]

where *S*_1_ (*S*_2_) is the signal proportional to the total amount of solid (liquid) and *F* is a pre-determined correction factor allowing for a dead-time in the receiver. Mixtures of MA in HOSO were warmed to 80 °C (left 15 min) and then cooled at 1 °C min^–1^ to set temperatures and left for 30 min before measurement. The results are given in Fig. 4, where it can be seen that the solid content increases with both a decrease in temperature (due to crystallisation) and an increase in the overall MA concentration (leading to more crystals). The mixtures are 100% liquid above 37 °C in agreement with the phase boundary determination in Fig. 1 (one-phase). Around room temperature of 22 °C where these solutions/dispersions have been aerated, the volume fraction of crystals present increases from *ca.* 0.1% at 4 wt% MA to *ca.* 5% at 12 wt% MA (taking the densities of both MA and HOSO around unity). These relatively low volume fractions of crystals (≤5%) are all that is required to form solid-like oil gels in which crystals interact with each other mainly through van der Waals forces.

### Aeration of crystal dispersions

#### Effect of MA concentration at 22 °C

**Fig. 5:**
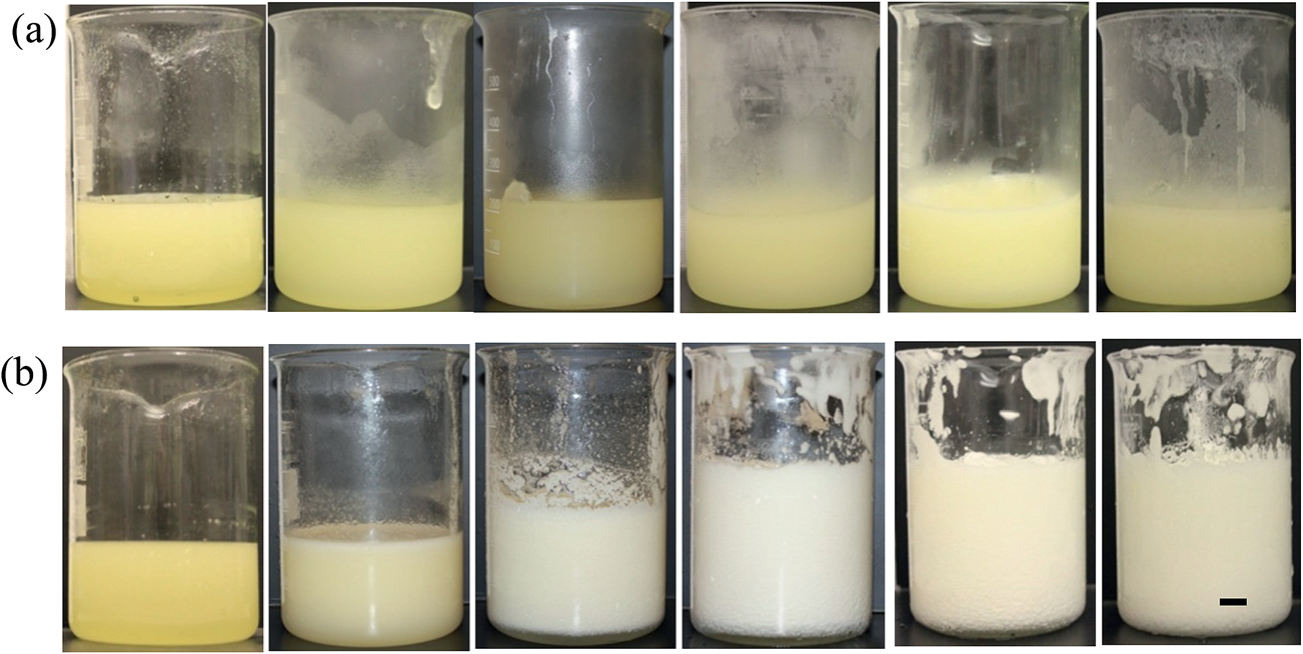
Photographs of vessels containing mixtures of MA in HOSO at different concentrations (a) before aeration and (b) immediately (within 1 min) after whipping at 22 °C. MA concentrations in wt% are (left to right) 2.0, 3.5, 4.5, 5.0, 6.0 and 10.0. The sample at 2.0 wt% is one-phase, all other samples are two-phase. Scale bar = 1 cm.

The aeration of MA solutions/dispersions in HOSO was carried out firstly as a function of MA concentration at 22 °C (route 1 in Fig. 1). For a consistent protocol, all mixtures (200 mL) were heated to 80 °C, cooled to 8 °C (freezer, 1 °C min^–1^) and allowed to warm gradually to room temperature (22 °C). They were then aerated with a double beater electric whisk for a total time of 45 min. During this time, for those mixtures capable of being foamed, the volume of foam increased with time (up to 15 min) and then remained constant. The appearance of the mixtures before aeration can be seen in Fig. 5(a) for selected MA concentrations (2–10 wt%). Below 2 wt% mixtures were clear. Between 2 and 3.5 wt% they were slightly turbid and fluid-like. Between 4 and 6 wt% they were turbid and gel-like, and above 7 wt% they were solid-like. Following aeration, Fig. 5(b) shows that foaming of these oil mixtures becomes possible but it is very dependent on MA concentration. At concentrations <3 wt% where the unaerated mixture is a molecular solution, no foam is formed. At and above 3.5 wt% where the unaerated mixture is two-phase containing crystals, foam can be produced whose initial volume increases progressively with MA concentration until reaching its maximum at and above 5 wt% MA. The foam volume decreases slightly at 12 wt% due to the difficulty of dispersing air bubbles in such a viscous gel. We note that the colour of the foam changes from yellow to white as the volume of air incorporated increases.

**Fig. 6:**
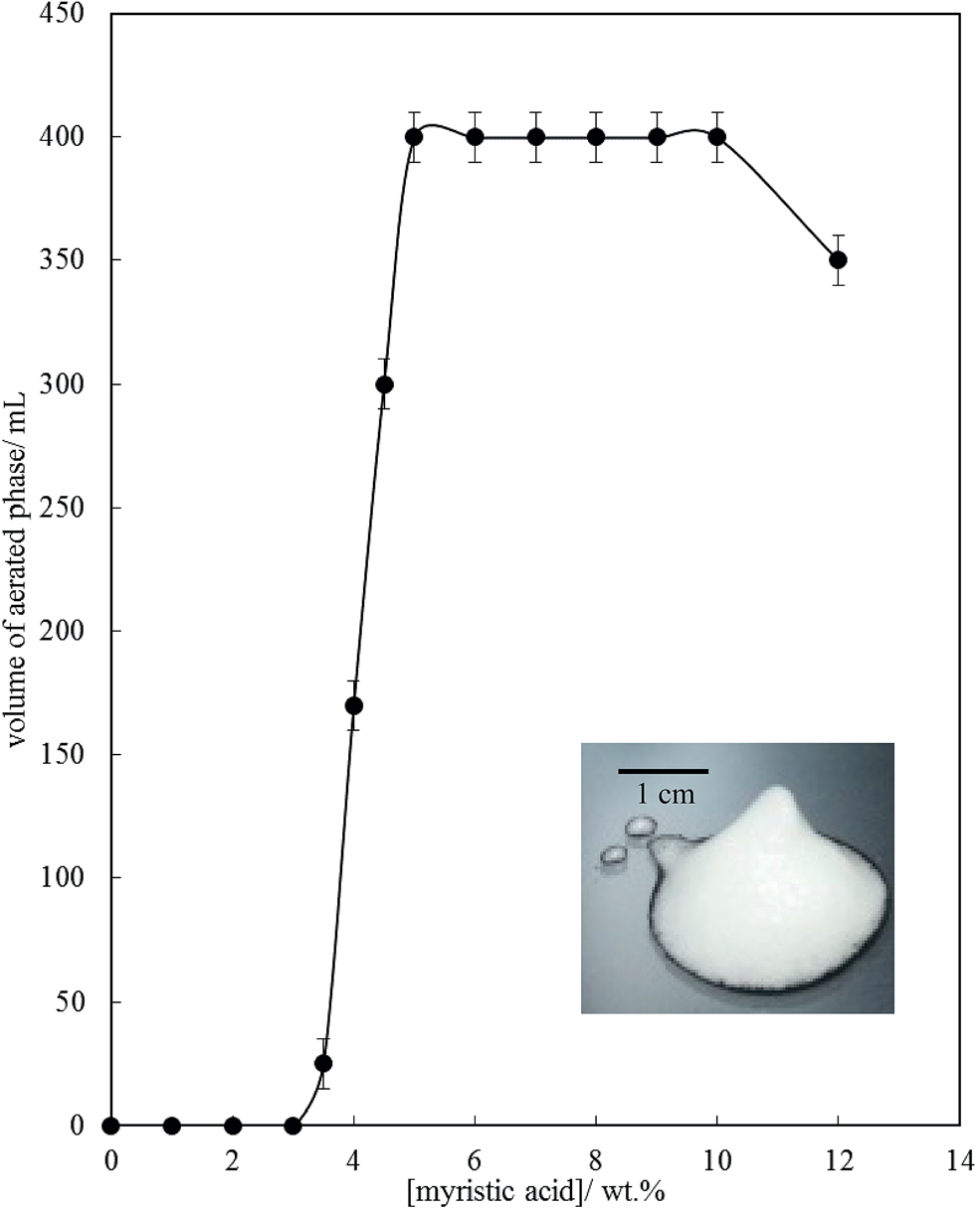
Volume of aerated phase (air + oil) produced after whipping 200 mL of MA–HOSO mixture at 22 °C as a function of the concentration of MA. Inset: photograph of a sample of the oil foam produced at 8 wt% MA.

Fig. 6 is a plot of the volume of the aerated phase as a function of MA concentration. In cases at lower concentration where not all the oil becomes incorporated into the aerated phase, we account for this in this parameter. In all other cases, the aerated phase contains all the oil and the incorporated volume of air. Once crystals are formed in the oil, foam can be stabilised and, as seen in Fig. 4, increasing the MA concentration at fixed temperature leads to an increase in the concentration of crystals (with little change in their size distribution) and a progressive increase in the foamability. The volume fraction of air in the oil foam increases from 0.22 ± 0.04 at 4 wt% MA to 0.52 ± 0.04 at 5 wt% MA and above, a value close to that reported in ref. 22 and close to the random close packing fraction of monodisperse spheres of 0.64. A photograph of a sample of foam prepared from 8 wt% MA is given in the inset to Fig. 6. It is thus likely that foams are stabilised by adsorption of MA crystals to the air–oil surfaces of bubbles and by the network of excess crystals in the oil phase creating a gel and impeding buoyancy-driven creaming of air bubbles within the foams. Whipping MA–HOSO mixtures in both the one-phase and two-phase regions has enabled us to determine whether molecular MA or particulate MA is responsible for foaming. We conclude that MA molecules are not surface-active enough at the air–HOSO interface to enable foaming and that MA crystals are surface-active at this interface. In order to cover as much of the air–oil interface as possible, it is likely that plate-like crystals of MA will orient with their faces parallel to the bubble surface. The driving force for their adsorption is to reduce the surface energy of the air–oil surface. In the case of alkane waxes in oil solvent, crystals consist of flat plates of alkane with large, slow growing flat faces exposing terminal methyl groups and small, fast growing edge faces which expose mainly methylene groups.^44^,^45^ The difference in surface energy between the low energy methyl-exposing surface and the higher energy methylene-exposing surface is approx. 8 mN m^–1^.^46^ For MA crystals, faces expose methyl groups and edges expose mainly methylene and carboxylic acid groups; we hypothesise that the faces are thus likely to be in contact with air (and oil) and the edges interact with each other through the carboxylic acid groups within the air–oil surface.

**Fig. 7:**
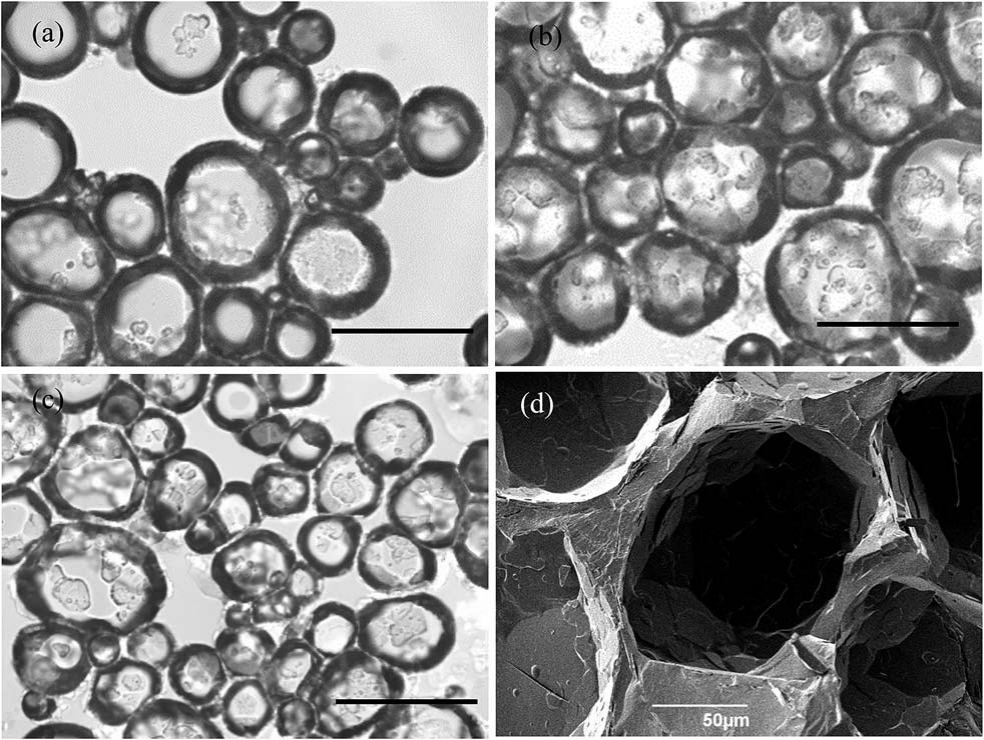
(a)–(c) Optical microscopy images of air-in-HOSO foams prepared at 22 °C and stabilised by crystals of MA at concentrations of (a) 5 wt%, (b) 7 wt% and (c) 8 wt%. Scale bars = 200 μm. (d) Cryo-SEM image of an oil foam formed at 22 °C containing 10 wt% MA.

Optical microscope images of the oil foams are given in Fig. 7(a–c) for three selected MA concentrations where crystals are formed. Most bubbles are non-spherical and possess a textured surface. This is indirect evidence that solid crystals are present at air bubble surfaces since, as witnessed in particle-stabilised aqueous foams,^47^,^48^ particles are compressed laterally within the surface and jamming occurs such that relaxation of bubble shape to spherical after foam formation becomes impossible. As a result of the high shear during foam formation, a polydisperse population of bubbles arises whose diameters vary between 50 and 200 μm. We cannot rule out the possibility that some crystals present in oil before whipping are broken to smaller ones and that these may adsorb preferentially to the smaller bubbles. The average bubble diameter is more or less the same for MA concentrations above 5 wt%, for which the volume fraction of air within foams reaches its maximum. Taken together, this implies that the fraction of non-adsorbed crystals, which increases with MA concentration, serve to strengthen the gel network in the continuous oil phase. Fig. 7(d) is a cryo-SEM image of the HOSO foam prepared at 10 wt% MA. Plate-like crystals of MA are clearly visible on the surface of the central air bubble viewed from within.

The stability of the oil foams at rest was monitored at 22 °C for up to 18 months. Their appearance immediately after formation and 6 months later can be seen in the photographs of Fig. S6† for smaller volume samples (30 mL HOSO). At the lower end of the MA concentration scale (≤6 wt%), approximately 30% of the initial oil volume drains below the foam, but all of this occurs within the first 24 h. At MA concentrations ≥8 wt% no oil drainage occurs since it is immobilised in the highly elastic oil gel. In addition, all of the foams showed no signs of bubble coalescence or disproportionation as evidenced by the very similar bubble size distributions compared with those determined initially, despite the increase in the dispersed phase volume fraction with time for the lower MA concentrations. The resistance of these two notorious instability mechanisms imparted by adsorbed MA crystals is remarkable. Such ultra-stable oil foams are in sharp contrast to those studied previously^9^–^13^ which only remained stable for up to a month or so.

#### Influence of temperature on foam stability

**Fig. 8:**
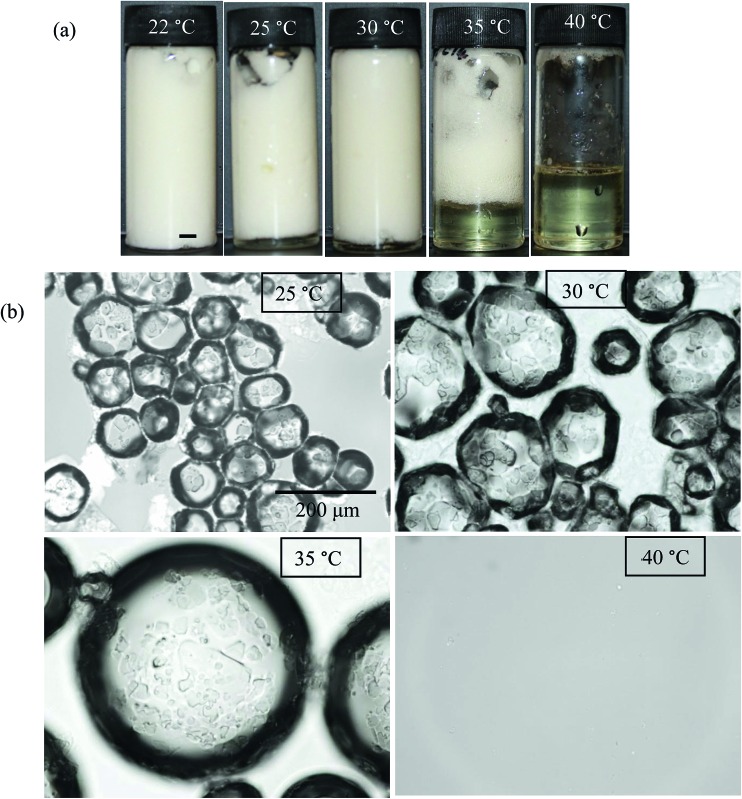
Effect of temperature on HOSO foams prepared at 22 °C and stabilised by 8 wt% MA. Heating rate was 1 °C min^–1^. (a) Appearance of vessels, scale bar = 1 cm and (b) optical micrographs of foams/solutions at temperatures specified.

Since molecular solutions of MA in HOSO crystallise on cooling and crystals formed melt upon heating, the possibility exists that oil foams stabilised by MA crystals should be responsive to changes in temperature leading eventually to complete foam destabilisation. Such stimuli-responsive foams are of interest industrially as they can be formed and destroyed quite easily and on demand. A similar idea was demonstrated for water-in-oil emulsions stabilised entirely by temperature-sensitive wax particles.^49^ HOSO foams were prepared at 22 °C and at different MA concentrations as described above. They were then heated in a water bath at around 1 °C min^–1^. Fig. 8(a) shows the appearance of the foam prepared at 8 wt% MA upon progressive heating. A completely stable foam at 22 °C collapses on warming by a combination of oil drainage and bubble coalescence such that by 40 °C no foam remains and a clear molecular solution of MA in HOSO re-forms. The effect of a temperature increase is twofold. Firstly, MA crystals originally adsorbed at air bubble surfaces melt and, as discussed above, MA molecules are not surface-active and so bubble coalescence ensues. Disproportionation by gas transfer through oil from smaller bubbles to larger ones is also likely once the stabilising layer of particles has been diluted or removed.^50^ Secondly, the oil gel present in the continuous phase is gradually destroyed as excess MA crystals melt such that its viscosity is reduced and oil drains. The concomitant compaction of the bubbles in the oil foam increases the possibility of coalescence and the foam is destabilised. By cooling the solution obtained at 40 °C to room temperature and re-whipping the resulting dispersion, stable oil foams were again formed demonstrating the reversibility of the process with respect to temperature. Microscope images of the foam obtained at the corresponding temperatures of Fig. 8(a) are given in Fig. 8(b). The progressive increase in the average bubble size between 25 °C (100 μm) and 35 °C (0.5 mm) is very noticeable even though crystals can still be seen on bubble surfaces at 35 °C. As expected, no bubbles are visible at 40 °C.

The time course of foam collapse with increasing temperature is shown in Fig. S7† for two concentrations of MA, where both the foam volume and the volume of drained oil are plotted. Oil drainage and foam reduction occur simultaneously and are initiated once the temperature reaches 27 °C. The temperature at which oil foams collapse completely increases with MA concentration, from 37.0 °C at 4 wt% to 41.4 °C at 12 wt%. This is in line with the increase in the dissolution temperature of oil dispersions with MA concentration seen in Fig. 1. We note however that foam collapse is complete at higher temperatures than the dissolution temperatures. It is anticipated that these temperatures will be dependent on both the foam preparation temperature and the rate of subsequent temperature increase.

#### Effect of aeration temperature at 8 wt% MA

**Fig. 9:**
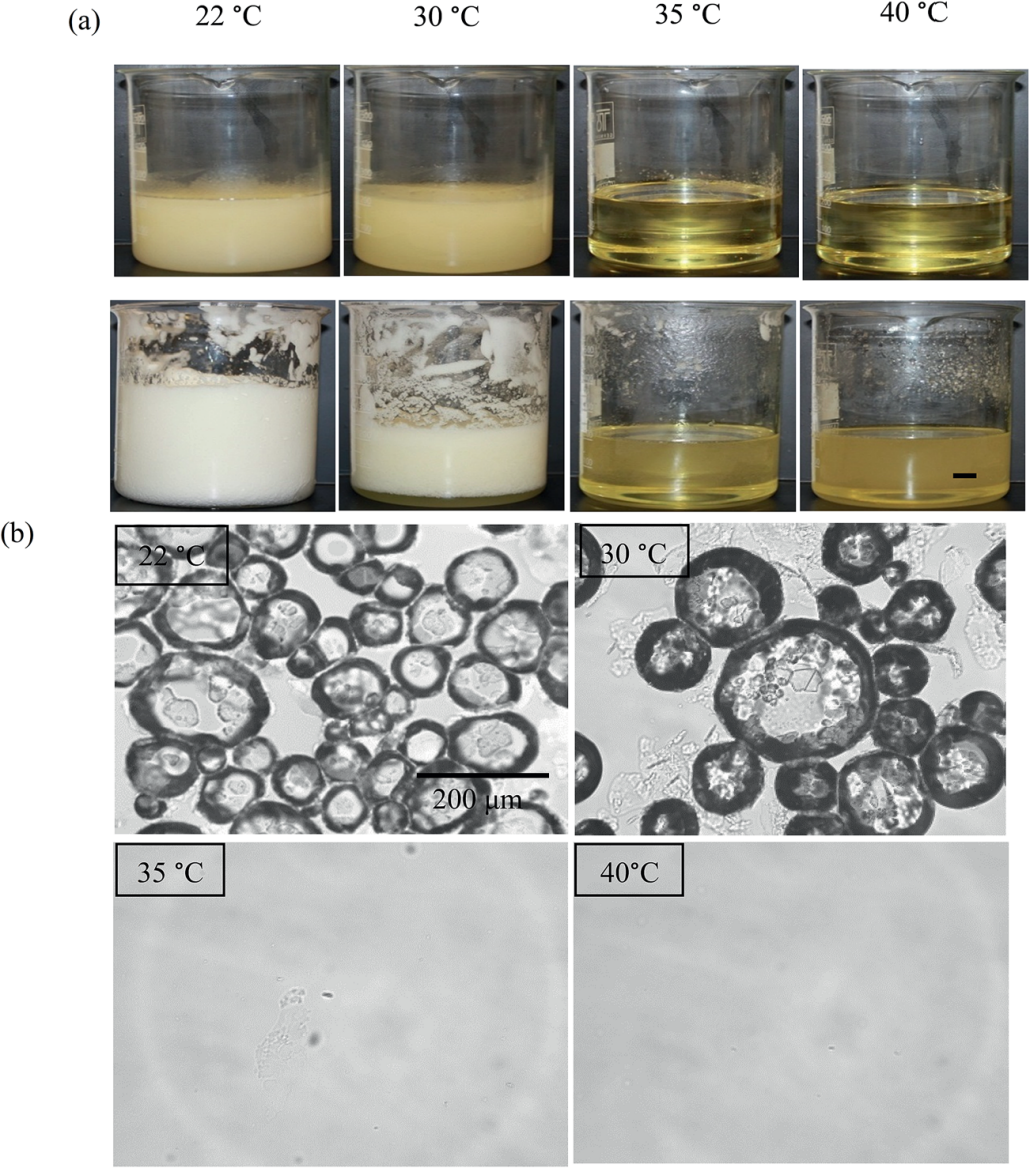
(a) Appearance of vessels containing 8 wt% MA in HOSO before (upper) and after (lower) whipping at different temperatures, scale bar = 1 cm and (b) optical microscope images of mixtures after whipping for temperatures given, scale bar same in all cases.

The aeration of MA solutions/dispersions in HOSO was also carried out at different temperatures at 8 wt% MA (route 2 in Fig. 1). Mixtures were first heated to 80 °C, cooled to 8 °C at 1 °C min^–1^ followed by slow heating to either 22, 30, 35 or 40 °C. Whipping with a double beater electric whisk was then carried out at these four temperatures. The appearance of the system before aeration is given in Fig. 9(a). The mixture was gel-like and turbid at the two lower temperatures, being less viscous at 30 °C *cf.* 22 °C. At the two higher temperatures, a clear, yellow liquid formed. We verified the presence of MA crystals in oil at 22 and 30 °C, and their absence at 35 and 40 °C (not shown). After whipping (Fig. 9(a)), a large volume of foam was produced at 22 °C, less foam formed at 30 °C and no foam was produced at 35 or 40 °C. Again, foam formation is only possible from MA crystal dispersions as opposed to MA molecular solutions, with crystals being sufficiently surface-active. If particles are wetted completely by either bulk phase (air or oil), the contact angle measured through oil is either 0° or 180° respectively. Values between these extremes imply that particles may adsorb at the air–oil surface. Using discs of compressed MA powder, the advancing contact angle through HOSO of a drop of HOSO in air was around 40° (*cf.* that for water of 89°), consistent with adsorption of MA crystals to air bubble surfaces in oil.

**Fig. 10:**
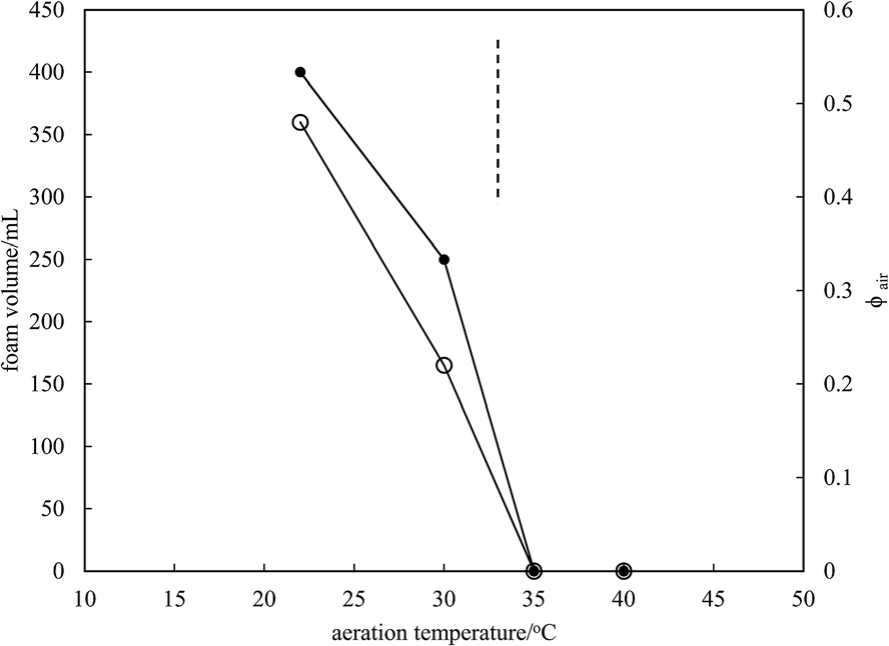
Variation of foam volume produced (filled points) and volume fraction of air in foam (unfilled points) *versus* aeration temperature for 8 wt% MA in HOSO. Mixtures originate from the two-phase region to the left of the dashed line and from the one-phase region to the right of it.

Microscopy images of the mixtures after whipping are seen in Fig. 9(b). A high density of bubbles appears at 22 °C with a lower density at 30 °C. Bubbles are non-spherical and have textured surfaces however at both temperatures due to the presence of adsorbed MA crystals. By contrast, no bubbles are observed in the mixtures whipped at 35 and 40 °C. The dramatic influence of the aeration temperature on the foam volume and the volume fraction of air incorporated into the foam is plotted in Fig. 10. It provides further proof that MA crystals are essential for foam stabilisation since, as discussed in Fig. 4, the solid content is zero for 8 wt% MA at temperatures >32 °C but increases to around 0.8% and 2.5% (interpolated) on lowering the temperature to 30 °C and 22 °C respectively. A relatively narrow temperature window of a few °C thus exists below which oil foams can be stabilised and above which they cannot.

#### Whipping of other vegetable oils

**Fig. 11:**
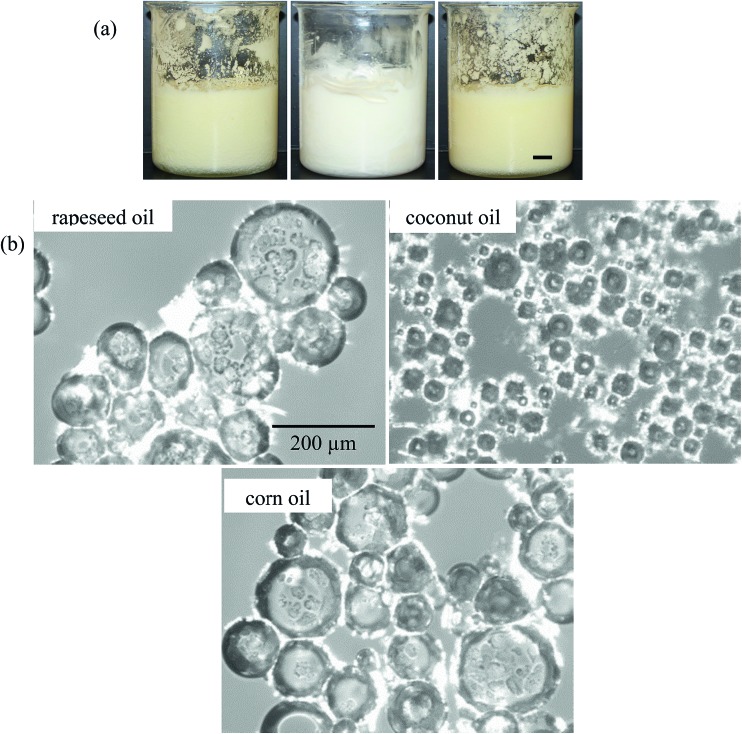
(a) Appearance of air-in-oil foams after whipping 5 wt% MA in oil at 22 °C for rapeseed oil (left), coconut oil (middle) and corn oil (right), scale bar = 1 cm and (b) corresponding optical micrographs, scale bar same in all cases.

In order to ascertain how general our findings with MA–HOSO mixtures are, we investigated the aeration of mixtures of 5 wt% MA (known to produce an oil gel in HOSO) in eight other vegetable oils at 22 °C. Including HOSO, all the chosen oils contain a high proportion of triglyceride possessing C_18_ chains (saturated or unsaturated) apart from cottonseed oil (mainly C_18_ and C_16_) and coconut oil (mainly C_14_ and C_12_).^51^ Apart from coconut oil which melts fully around 24 °C, their melting points are below 0 °C. This means that they remain liquid at the lowest temperature reached (8 °C) after being placed in the freezer. After cooling from 80 °C to 8 °C and warming to 22 °C, an oil gel was formed in all the oils. Coconut oil is an interesting example as it is semi-solid at 22 °C and so MA crystals are dispersed in a semi-solid fat matrix. Needle-like or plate-like crystals were observed in the gels by microscopy. A foam could be produced in all oils but Table 2 shows that the volume fraction of air incorporated and the whipping time required to produce maximum foam volume varied considerably for the different oils. Some oils, *e.g.* olive, could be whipped quickly (5 min) but were not very foamable (*φ*_air_ = 0.26) whereas other oils, *e.g.* HOSO, were more foamable (*φ*_air_ = 0.54) but took longer to aerate (15 min). The visual appearance of selected foams of rapeseed, coconut and corn oil can be seen in Fig. 11(a) alongside their corresponding optical micrographs in Fig. 11(b). Non-spherical bubbles possessing textured surfaces are apparent, with particularly small bubbles (average diameter *ca.* 50 μm) stabilised in coconut oil. It may be that in this case, crystals of MA and low chain triglycerides (from the oil) coat air bubbles dispersed in a semi-solid fat continuous phase. The stability of the foams after 24 h is summarised in Table 2. Not surprisingly, no ‘oil’ drainage occurs with the coconut oil foam (since semi-solid); the foams formed in the liquid oils display widely varying stability to both coalescence and drainage. Thus, those of peanut and cottonseed disappear completely whereas those of HOSO and corn are the most stable. Immediately after preparation at 22 °C, foams were heated at 1 °C min^–1^ as before to determine their temperature responsiveness. They were all rendered unstable upon warming with the temperature required for complete foam breakdown varying by over 10 °C from 32.3 °C to 43.7 °C. Although we have not studied the behaviour in each oil in detail, we suspect that the differences in foam properties highlighted in Table 2 will be related to where in the solubility–temperature diagram this composition falls (5 wt% MA, 22 °C). This is largely determined by the fatty acid type and composition within the triglyceride chains of the oil molecules. We thus show that adsorption of MA crystals to air–oil surfaces yielding stable oil foams can be achieved in a variety of vegetable oils containing different kinds and compositions of triglyceride molecules and these foams are also temperature responsive.

**Table 2:** Characteristics of air-in-vegetable oil foams stabilised by 5 wt% MA and prepared at 22 °C. The oils are liquid at this temperature apart from coconut which is semi-solid (fat, end of melting range ≈ 24 °C). For stability, % are those relative to the total foam + oil volume

Oil	*φ*_air_	Whipping time for maximum foam/min	Appearance after 24 h	Temperature for complete foam collapse/±0.1 °C
HOSO	0.54	15	Foam (75%) + drained oil (25%)	37.0
Rapeseed	0.43	5	Foam (43%) + drained oil (57%)	41.8
Coconut	0.34	15	Foam only (100%)	32.3
Soybean	0.33	15	Foam (57%) + drained oil (43%)	39.4
Corn	0.32	10	Foam (67%) + drained oil (33%)	43.4
Olive	0.26	5	Foam (40%) + drained oil (60%)	43.7
Peanut	0.22	5	Total foam collapse	42.4
Cottonseed	0.21	10	Total foam collapse	41.3
Sesame	0.17	5	Foam (20%) + drained oil (80%)	42.4

## Conclusions

We describe a method for preparing air-in-vegetable oil foams stabilised by crystals of fatty acid. The crystals are formed *in situ* on cooling a molecular solution at high temperature to form a crystal dispersion at low temperature. Foaming is only possible once crystals are formed. The foams are very stable to drainage, coalescence and coarsening due to a combination of solid crystals at air bubble surfaces and gelling of the continuous oil phase with excess interacting crystals. The foams are temperature-sensitive and destabilise completely on warming to around the melting point of the crystals; stable foams can be prepared again by subsequent cooling and aeration demonstrating the reversibility of the process. The method is relatively simple and can be extended to a wide range of oil-soluble surfactants of different structure which are cheap and readily available.

## Experimental

### Materials

The main triglyceride oil was high oleic sunflower oil or HOSO, batch 002/12 purchased from Eulip (Italy). Its fatty acid composition is 84% oleic (C_18_, one C=C bond), 12% palmitic and stearic mixture (C_16_/C_18_, saturated) and 4% linoleic (C_18_, two C=C bonds). It was passed twice through a basic alumina column to remove polar impurities before use. It exhibits excellent chemical stability without hydrogenation. HOSO is a clear, slightly amber-coloured liquid of density 0.92 g cm^–3^ and surface tension equal to 33.4 mN m^–1^, both at 25 °C. Myristic acid, MA, is a saturated C_14_ carboxylic acid and was 99% pure from Acros Organics. Air was that within the laboratory (78% N_2_, 21% O_2_, 1% rare gases/CO_2_). The other vegetable oils from Acros Organics were commercial grade and included rapeseed (BCBJ4393V), coconut (A0338345), sesame (A0338971), soybean (MKBN8800V), cottonseed (A0338268), corn (A0341121) and olive (A0335272). Peanut oil was also from Eulip (50784).

### Methods

#### Determination of solubility of MA in HOSO

A total of 1 g of a mixture of MA in HOSO contained in a 37 mm × 10 mm glass vial with a screw cap was placed in the well of a Grant GD120 thermostat. A thermocouple was used to monitor the temperature of a separate sample of HOSO in the same water bath. The temperature was first set at 80 °C for 10 min, resulting in a clear homogeneous liquid at all MA concentrations. The mixture was then cooled at a rate of 0.1 °C min^–1^. When the first crystal formed in bulk, we recorded this as the onset of crystallisation. The sample was cooled until 5 °C below complete crystallisation (when turbidity remained constant) then held for 5 min at this temperature. The sample was then warmed at 0.1 °C min^–1^ until it was clear again. The dissolution temperature was taken as the temperature at which the sample started to clear or became less turbid. Although this is more subjective, it makes more sense to consider the onset of melting as we originally determined the onset of crystallisation.

#### Differential scanning calorimetry

Mixtures of MA in HOSO were studied using a Perkin Elmer Differential Scanning Calorimeter 7 (DSC) instrument. The samples were placed in an aluminium sample pan with an empty sample pan as a reference. Indium was used to calibrate the instrument. The samples were cooled from 80 °C to –20 °C, held for 5 min at this temperature and then heated to 80 °C. Heating and cooling rates of 2 °C min^–1^ and 20 °C min^–1^ were investigated.

#### X-ray diffraction

X-ray diffraction was used to identify the crystal structure of MA in a 10 wt% MA in HOSO gel. A PANalytical Empyrean instrument with Highscore Plus processing software was used employing a CuKα source (*λ* = 1.5406 Å) and the 2*θ* angles investigated were 2° to 50° with a step size of 0.02°. An additional X-ray diffractometer was used to identify the crystal structure of an aggregate of myristic acid extracted from the same gel. This was a STOE X-ray IPDS2 diffractometer with MoKα radiation of 0.7107 Å with the 2*θ* angles investigated ranging from 2° to 50°. The temperature of the investigated samples was 20 ± 2 °C.

#### Optical microscopy

Various samples including MA solutions or gels in vegetable oil and oil foams were placed on a glass microscope slide with a cover slip and observed using an optical microscope (Leica DME). The microscopic analysis was carried out with and without cross polarising lenses. Images were captured with a Leica DFC 290 camera and analysed using a Leica V3 application suite calibrated with a PYSER-SGI limited graticule. The temperature of the slide was controlled with a Linkam PE120 heat stage attached to a Linkam T95-PE processor.

#### Cryo-scanning electron microscopy (SEM)

Oil foams of HOSO stabilised by MA crystals were analysed using cryo-SEM. A sample of the foam was added to a perforated aluminium stub and plunged into liquid nitrogen slush (–210 °C). The frozen sample was then transferred to a cryo system (Quorum Technologies PP3010T), where it was held at a pressure of 10^–6^ mbar and at –140 °C in a nitrogen gas atmosphere. The sample was sputter coated with platinum. The sample then entered the SEM chamber of an EVO 60 microscope at a temperature of –140 °C and pressure of 10^–6^ mbar at a beam voltage of 15 kV and probe current of between 20 and 35 pA. The images were collected at various locations within the sample.

#### Contact angle determination

A 0.05 mL drop of either water or HOSO was added to the surface of a myristic acid pellet (thickness 2 mm, diameter 15 mm). The advancing and receding contact angle in air was measured through the liquid phase with the use of a Krüss DSA10 apparatus. Up to 20 measurements were taken. The pellet was produced by grinding MA into a fine powder with a pestle and mortar, then compressing the powder with the use of a Perkin Elmer hydraulic press with a weight of 12 tons.

#### Rheology

Rheological measurements of oil solutions/gels were conducted with a Physica MCR301 Anton Parr rheometer. For oscillatory rheology (with 0.01% strain and 1 Hz) we used a parallel plate geometry where approximately 0.5 g of the sample was added to the lower plate which had a Peltier system for temperature control. The distance between the plates (the sandblasted upper plate was 50 mm in diameter) was 0.5 mm. The non-aerated oil mixtures were added to the rheometer at 80 °C. The temperature of the sample was then decreased at a rate of 1 °C min^–1^ to 10 °C.

#### Pulsed nuclear magnetic resonance

The solid content of the MA–HOSO mixture was investigated with the use of pulsed NMR (Maran 25 resonance instrument) with RINMR software. The measurements were conducted by heating mixtures to 80 °C, holding the sample for 15 min and then lowering the temperature at 1 °C min^–1^ to 0 °C. The solid content was measured at 50, 45, 40, 37.5, 35, 32.5, 30, 25, 20, 10 and 0 °C. The sample (approx. 2 g in an NMR tube) was at the specified temperature for 30 min before the solid content measurement was conducted.

#### Whipping of MA–HOSO mixtures

The whipping technique involves mixing the MA–oil mixture (200 mL in a 250 mL glass beaker) at 80 °C for 1 h to create a clear, homogeneous solution. The mixture was then transferred to a 600 mL glass beaker and cooled to 8 ± 2 °C at approximately 1 °C min^–1^ by placing it in a freezer (Bio cold lab.) for 1 h (internal temperature –15 °C) and allowing the sample to warm naturally to 22 °C. This was followed by whipping with a hand held double beater electric whisk (Argos value range, blade size 6 cm) on speed setting 1 for a total of 45 min (5 min followed by rest interval of 5 min, repeat). After each 5 min duration of whipping, ∼1 mL of sample was placed onto a microscope slide with 1 mL of oil along with a coverslip and analysed with a microscope (Leica DME). If after 45 min of whipping a whipped oil was produced, the stability of the whipped product was then investigated by keeping a sample (30 g) at room temperature (22 ± 2 °C) and also heating a sample (10 g). The stability of the whipped product to an increase in temperature was investigated by placing 10 g of the foam into a 71 mm high by 26 mm wide glass bottle which was in the well of a Grant thermostat with a thermometer inside and heated at 1 °C min^–1^. Microscopy of the whipped product was undertaken at different temperatures.

## Supplementary Material

Supplementary information
