# Supplementary material for: Whipped oil stabilised by surfactant crystals
Source: Chem Sci. 2016 Mar 3;7(4):2621–32. doi: 10.1039/c6sc00046k (PMC5477051; doi:10.1039/c6sc00046k)

Electronic Supplementary Material (ESI) for Chemical Science.
This journal is © The Royal Society of Chemistry 2016

ESI

WHIPPED OIL STABILISED BY SURFACTANT CRYSTALS

Bernard P. Binks,[1,*] Emma J. Garvey[1] and Josélio Vieira[2]

[1] *Department of Chemistry, University of Hull, Hull. HU6 7RX. UK*

[2] *Nestlé Product Technology Centre, PO Box 204, Haxby Road, York. YO91 1XY. UK*

Figure S1. Variation of (a) precipitation temperature and (b) dissolution temperature with MA concentration in mixtures with HOSO for different rates of temperature change (given). The data are from visual observations at 0.1 ºC/min and from DSC curves for the two higher rates.

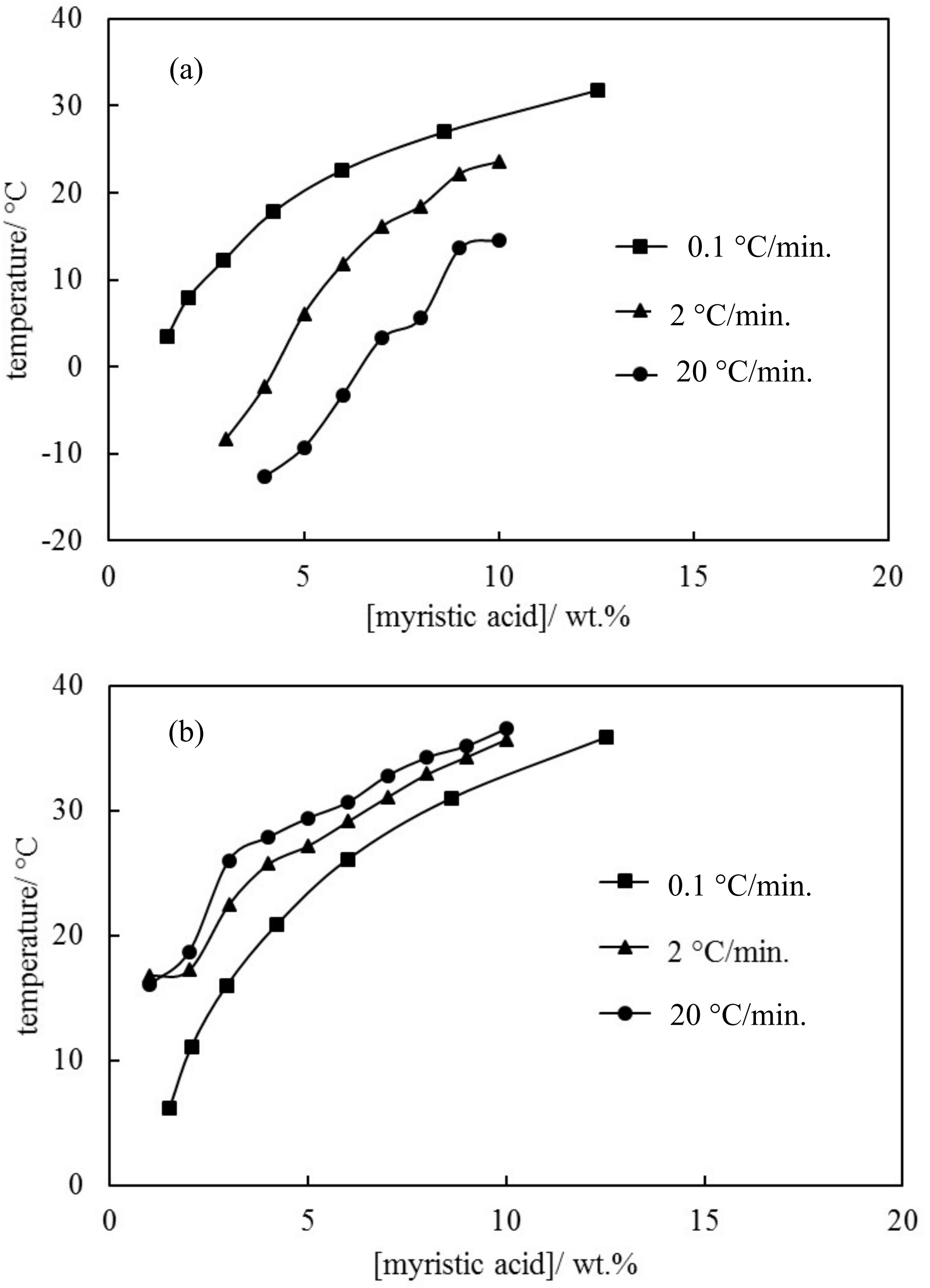

Figure S2. XRD spectrum of a 10 wt.% MA in HOSO gel using CuKα radiation (λ = 1.5406 Å).

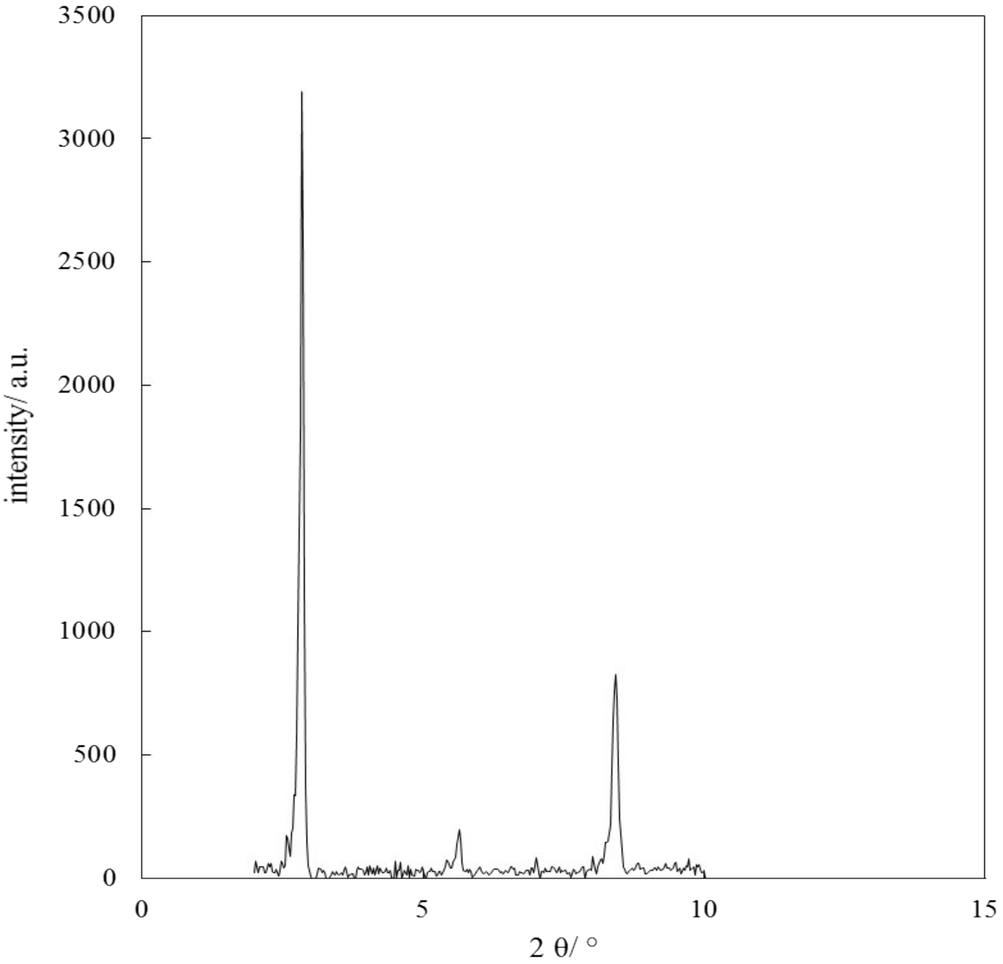

Figure S3. (a) Microscope image of the crystalline aggregate extracted from a gel containing 10 wt.% MA in HOSO and dried on filter paper. The plate-like aggregate at the centre of the image is mounted on a glass fibre, (b) X-ray diffraction data collected from (a) using MoKα radiation (λ = 0.7107 Å). The d-spacings for each ring are marked on the figure. The image is for a single 60 min exposure. The crystal was centred within the beam and rotated during data collection.

(a)

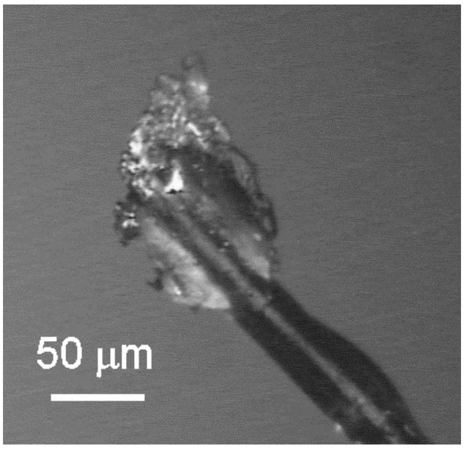

(b)

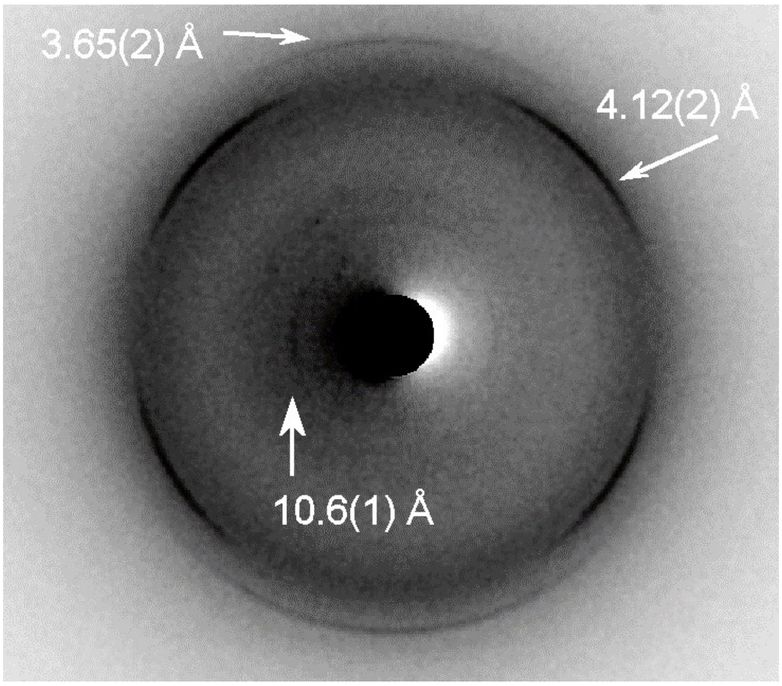

Figure S4. (a) Variation of G´ with frequency for MA in HOSO gels at 10 ºC at three concentrations. The strain was fixed at 0.01%. (b) Variation of G´ and G´´ with temperature for MA in HOSO gels at higher concentrations (given). Measurements were conducted at a strain of 0.01% and a frequency of 1 Hz.

(a)

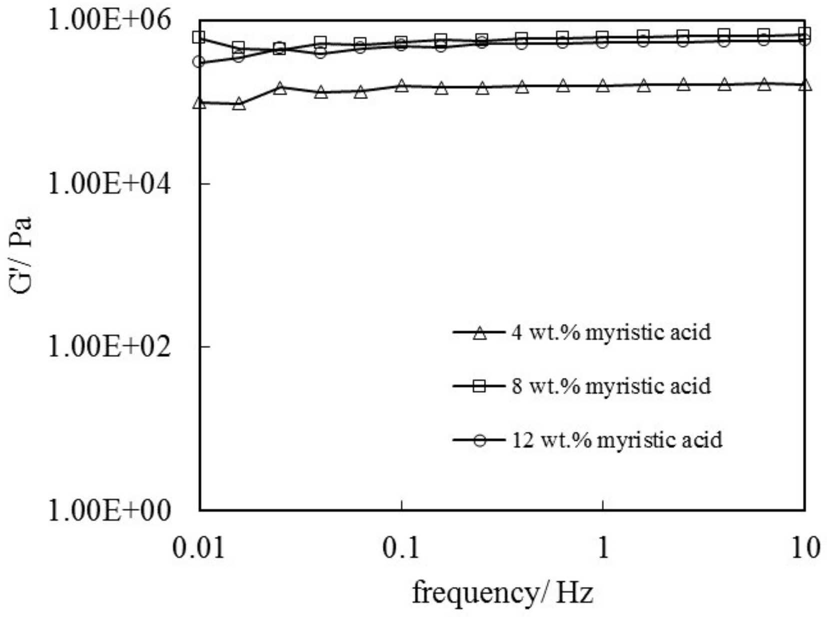

(b)

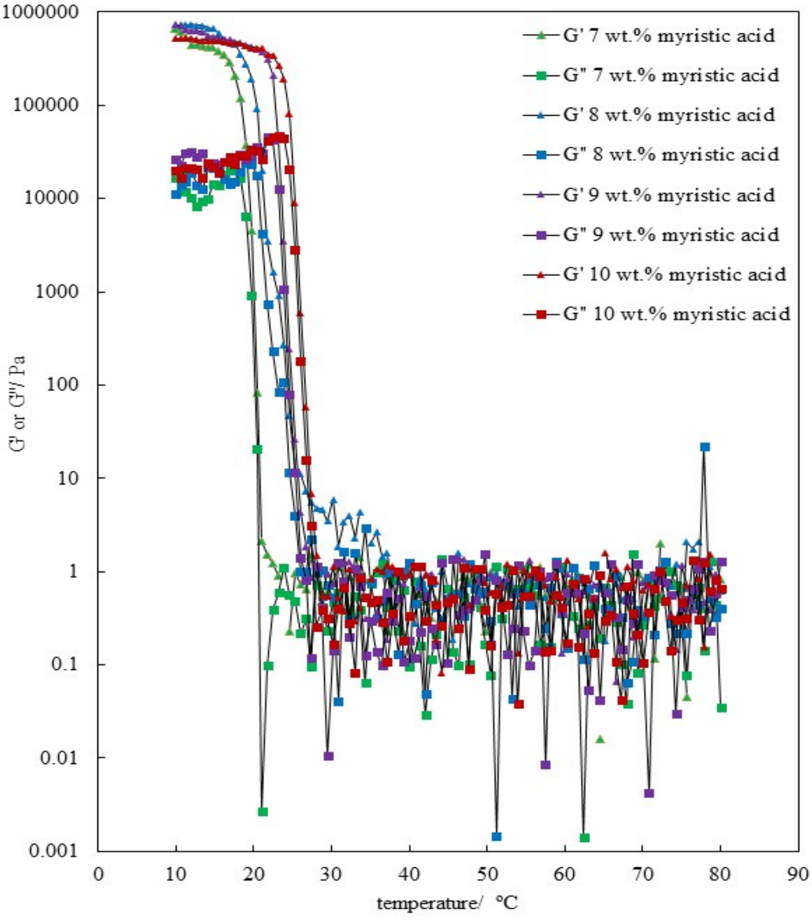

Figure S5. Variation of the precipitation temperature (visual), gelation temperature (midway between min. and max. G´) and temperature at which G´ is maximum with MA concentration in HOSO gels. Visual observations refer to a cooling rate of 0.1 ºC/min, those of rheology were at 1 ºC/min.

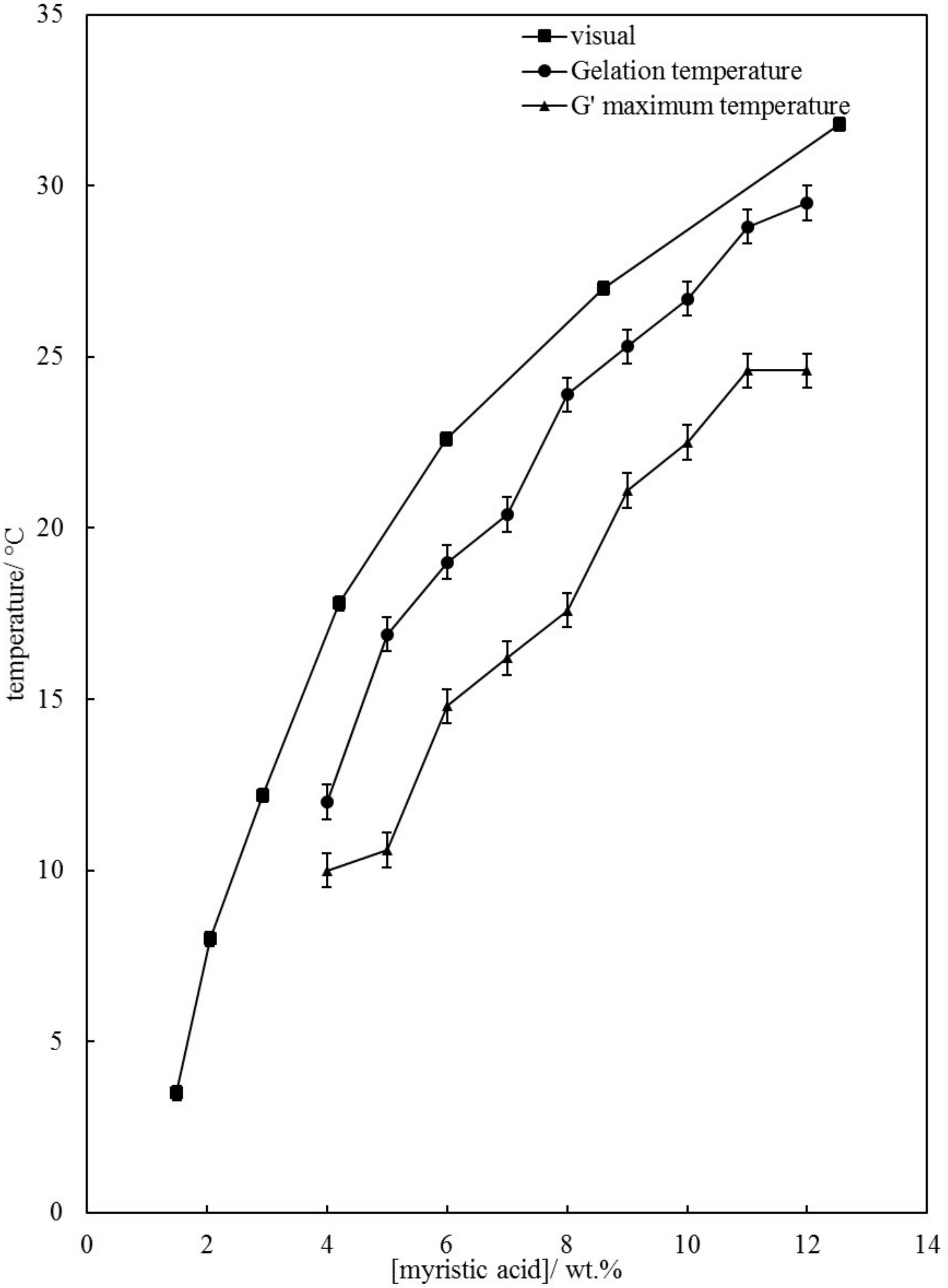

Figure S6. Photographs of vessels containing air-in-HOSO foams stabilised by MA at different concentrations (a) immediately after whipping at 22 ºC and (b) 6 months later. Scale bar = 1 cm.

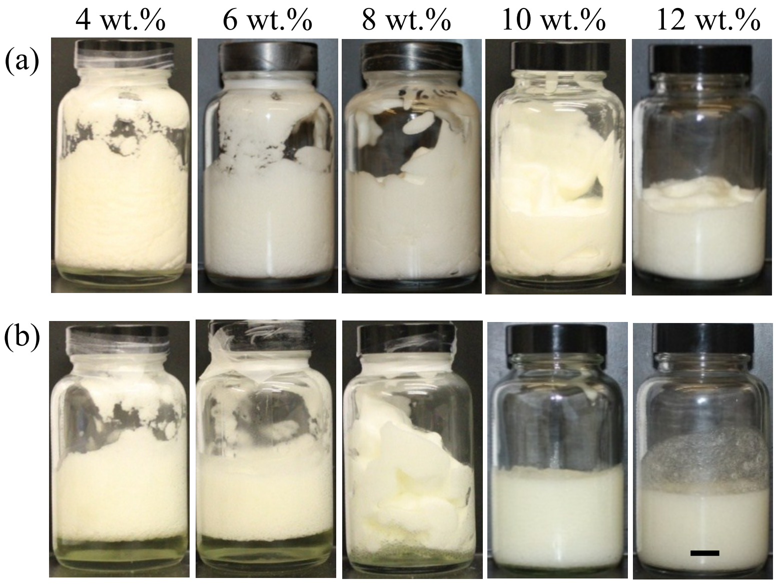

Figure S7. Volume of oil drained (filled points) and volume of foam (unfilled points) following heating at 1 ºC/min of an air-in-HOSO foam stabilised by either 5 wt.% or 10 wt.% MA prepared at 22 ºC.

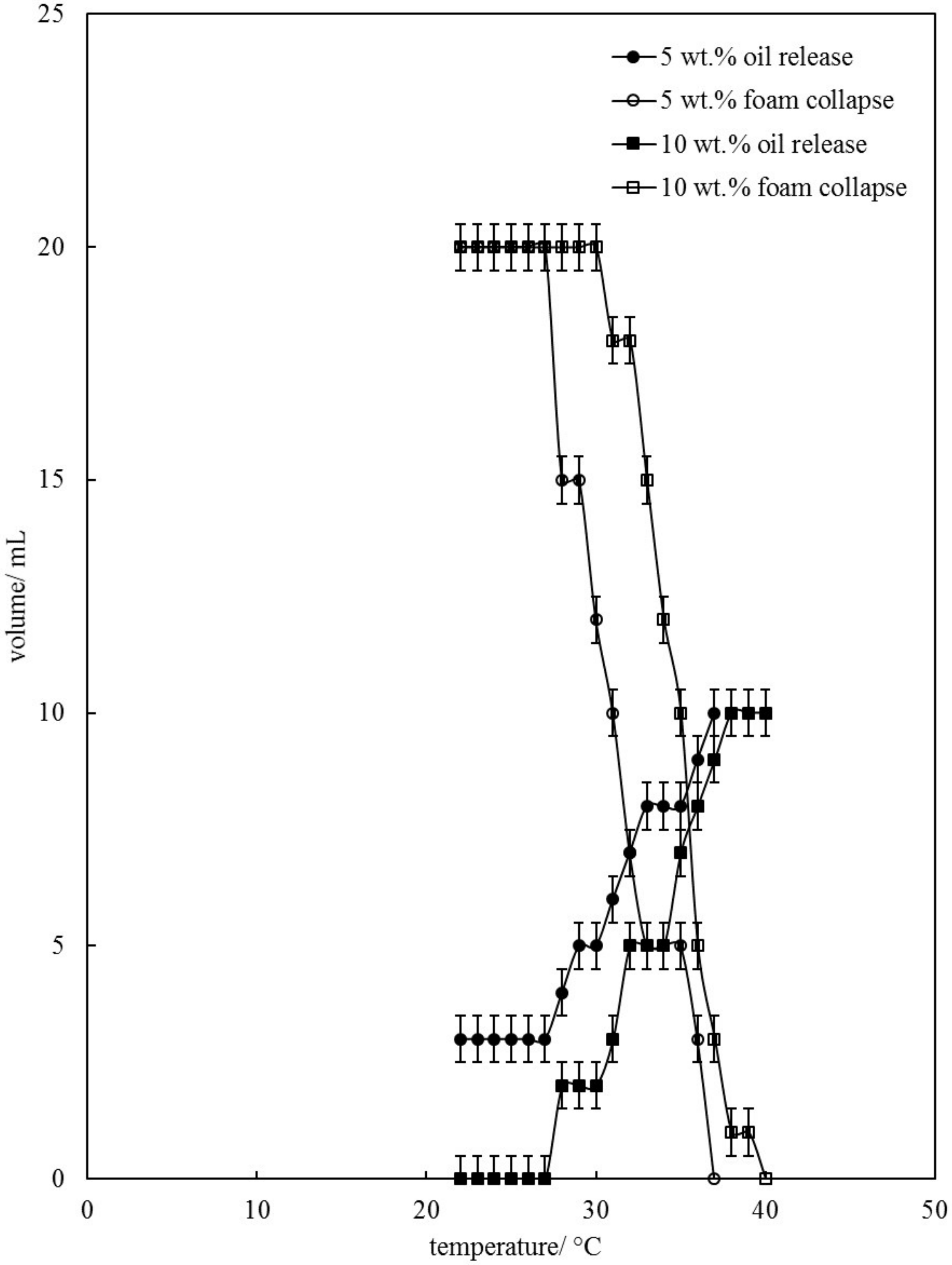

Supplement: Supplementary file 1 [file SC-007-C6SC00046K-s001.pdf]
